# Action affects perception through modulation of attention

**DOI:** 10.3758/s13414-021-02277-2

**Published:** 2021-03-15

**Authors:** Wladimir Kirsch, Tim Kitzmann, Wilfried Kunde

**Affiliations:** Department of Psychology, University of Würzburg, Würzburg, Germany

**Keywords:** Attention, Perception, Action

## Abstract

The present study explored the origin of perceptual changes repeatedly observed in the context of actions. In Experiment 1, participants tried to hit a circular target with a stylus movement under restricted feedback conditions. We measured the perception of target size during action planning and observed larger estimates for larger movement distances. In Experiment 2, we then tested the hypothesis that this action specific influence on perception is due to changes in the allocation of spatial attention. For this purpose, we replaced the hitting task by conditions of focused and distributed attention and measured the perception of the former target stimulus. The results revealed changes in the perceived stimulus size very similar to those observed in Experiment 1. These results indicate that action’s effects on perception root in changes of spatial attention.

**Supplementary Information:**

The online version contains supplementary material available at 10.3758/s13414-021-02277-2.

Whether and how perception changes in the context of actions has been examined in diverse studies in the past decades, and many different effects have been reported (see, e.g., Harris et al., 2015; Hommel et al., 2001; Proffitt, & Linkenauger, 2013; Witt, 2011; Zwickel & Prinz, 2012, for reviews; and see Firestone & Scholl, 2016; Philbeck & Witt, 2015, for controversies). For example, the perceived size of target objects proved to increase with an increase in success of planned or executed actions directed to these objects (Cañal-Bruland & van der Kamp, 2009; Cañal-Bruland et al., 2011; Cooper et al., 2012; Gray, 2013; Gray et al., 2014; Lee et al., 2012; Wesp et al., 2004; Witt & Dorsch, 2009; Witt et al., 2008; Witt & Proffitt, 2005). This outcome seems to correspond well with the subjective reports of athletes in many sports (cf. Witt & Proffitt, 2005). Here, we suggest that the origin of this and related phenomena is closely linked to changes in the distribution of spatial attention.

There is strong evidence that attention alters appearance of several object features, such as location (Suzuki, & Cavanagh, 1997), shape (Fortenbaugh et al., 2011), contrast (Liu et al., 2009), or spatial frequency (Gobel & Carrasco, 2005; see also Carrasco & Barbot, 2019, for a review). One observation is particularly relevant for the present report. When attention is focused at the center of a peripheral target stimulus, that stimulus is perceived as larger than in a neutral attention condition (Anton-Erxleben et al., 2007; Kirsch et al., 2018; Kirsch et al., 2020). Importantly, an increase in the size of the attentional focus decreases the perceived size of the stimulus (Kirsch et al., 2018). Assuming that attention is more focused at target objects in successful than in unsuccessful actions would predict several findings mentioned in the previous paragraph (see also Cañal-Bruland et al., 2011; Gray, 2013; Gray & Cañal-Bruland, 2015, for a similar suggestion). Accordingly, focusing attentional resources at a target object should be advantageous for action performance (e.g., Castaneda & Gray, 2007).

Moreover, this link of action-related influences on perception to attention could also resolve some related, but at first glance discrepant, results. It has been repeatedly reported that successful actions increase the perceived size of aimed target objects as mentioned. For example, successful golfers judged golf holes as being larger than less successful golfers do (Witt et al., 2008), successful football players judged the goal posts to be wider apart (Witt & Dorsch, 2009), successful archers judged the target to be bigger (Lee et al., 2012). These and related observations were taken as evidence that perception is scaled according to action ability of the observer (Proffitt & Linkenauger, 2013; Witt, 2011). In one of our earlier studies, we tested this claim using a computerized hitting task (Kirsch et al., 2014). Participants sat at a table with their hand holding a stylus on a digitizing tablet. Visual stimuli were projected on the plane of the tablet while the vision of the arm was prevented. The task was to hit a circular target by stylus movements starting at a varying distance to the target and controlling a visual cursor that disappeared in the course of the movement. In contrast to the aforementioned studies, we observed a negative relation between action success and judged target size. That is, participants whose hitting performance was relatively good, tended to estimate the target as smaller than did participants whose hitting performance was relatively bad (Experiments 1 and 2). Moreover, a larger movement distance that went along with weaker hitting performance was associated with a larger estimate of target size (esp. Experiment 3). These findings cannot be easily reconciled with a direct impact of action ability or action success on perception. However, if the participants’ attention was more focused with less successful actions in our study (see also p.1762 in Kirsch et al., 2014), then the apparent discrepancy between our and previous results can be resolved. This could be because conditions that are experienced to be more difficult require more effort to hit the target, resulting in a more focused mode of attention.^1^ The goal of the present study was to test this hypothesis.

Two experiments are reported below. In Experiment 1, we conceptually replicated the effect of the varying movement distance on perceived target size using a new version of the previous hitting task. In Experiment 2, we then varied the size of the attentional focus under comparable experimental conditions. The results of both experiments were very similar, indicating that the perceptual effect observed in Experiment 1 and in one of our earlier studies in the context of actions roots in changes of spatial attention.

## Experiment 1

The goal of Experiment 1 was to conceptually replicate an increase in perceived target size with an increase in movement distance observed in the hitting task of Kirsch et al. (2014). An increase in movement distance can be assumed to decrease the ability to hit the target in accordance with Fitts’ law (Fitts, 1954). This effect can thus be construed as a proxy for a series of related observations indicating changes in the perception of target objects aimed by actions following changes in action ability (or in action success; see Introduction).

Participants aimed to hit a circular target stimulus with a stylus movement under restricted feedback conditions. In contrast to our previous study, stimuli were now presented in the fronto-parallel plane (i.e., not in the plane of stylus movements). During movement planning, the apparent size of the target was measured by a method of constant stimuli. More specifically, we now adopted an approved protocol that was previously used in the research on visual appearance (Carrasco et al., 2004; see also Carrasco & Barbot, 2019). An additional circular stimulus was presented peripherally to the target stimulus and the task was to judge which of both stimuli is larger. One of the stimuli served always as a standard stimulus, the other as a test stimulus. The rationale was as follows: When the central stimulus is perceived differently in one compared with another condition, then shifts in the psychometric function of opposite direction should be observed depending on whether a standard or a test stimulus were centrally presented.

The critical experimental manipulation concerned the distance of the planned stylus movement. Based on our previous results we expected to find an increase in perceived target size with an increase in planned movement distance (Experiment 3 in Kirsch et al., 2014). Please note that the distance variation was rather small, so that a perceptual effect of only small magnitude could be expected.

### Methods

#### Participants

Sixteen volunteers participated in Experiment 1. The sample included 11 females and five males (*M* = 26 years, *SD* = 6). All participants reported to have normal or corrected-to-normal vision and to be right-handed. They received course credit (one participant) or monetary compensation (see below) for their participation. The sample size was determined a priori based on prior research and ensured a power of 1 − β = 0.95 for effect sizes of *d*_z_ = 0.89 (as estimated from Experiment 3 of Kirsch et al., 2014). The study was conducted in accordance with the ethical guidelines (2016) of the German Psychological Society (DGPs) as well as with Declaration of Helsinki.

#### Apparatus

**Fig. 1:**
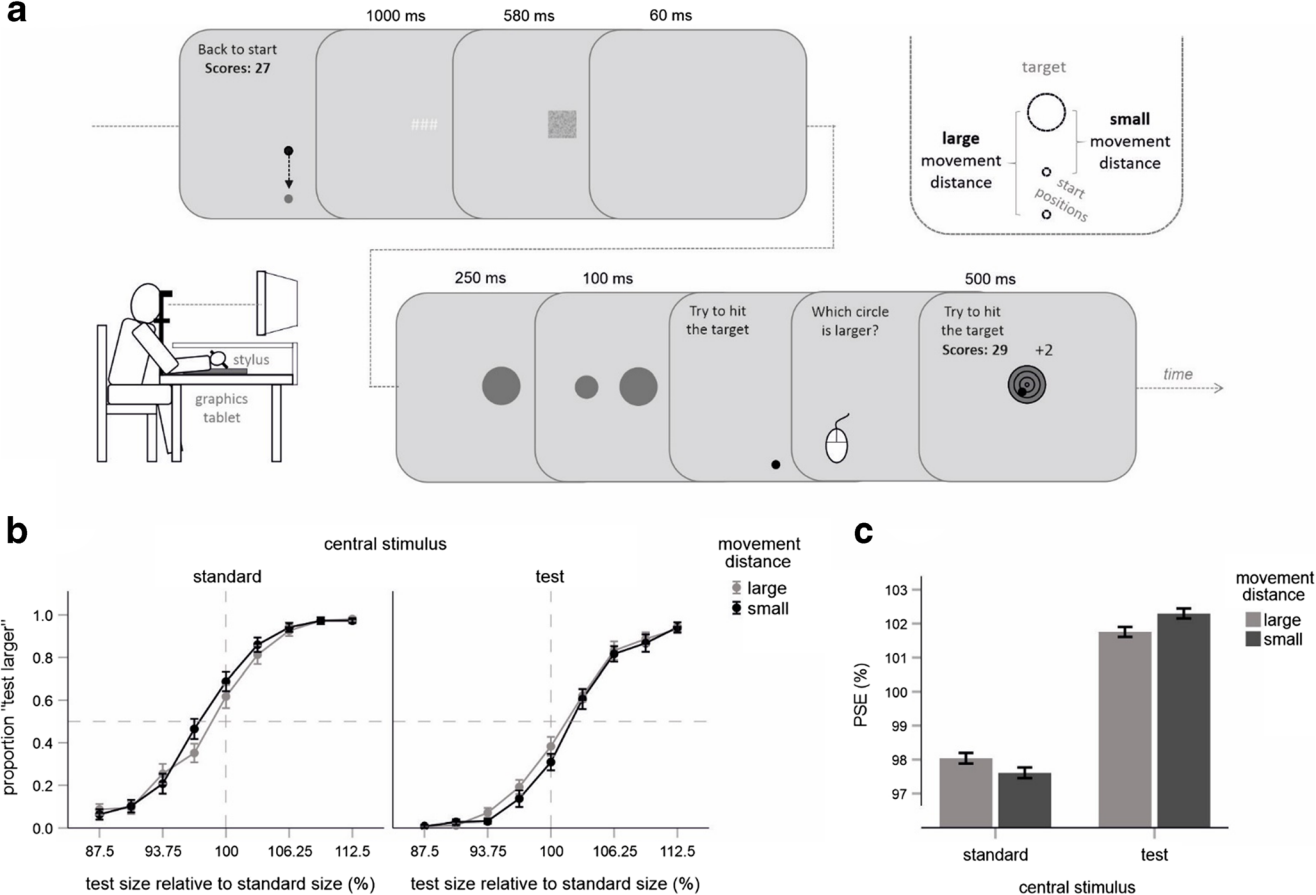
Experiment 1. **a** Main trial events, experimental setup (lower left part), and the critical variation of movement distance (upper right part). Stimuli are not drawn to scale. **b** Mean proportions of “test larger” judgments as a function of the type of central stimulus, movement distance, and of the size of the test stimulus. Error bars are standard errors indicting the variability across participants. **c** Mean PSE values as a function of the type of central stimulus and of movement distance. Error bars indicate within-participants standard errors computed according to Cousineau (2005)

The experiment was performed in a dimly lit room. Stimuli were displayed on a 19-in. CRT monitor (Samsung Samtron 96B; 100 Hz refresh rate; 1,024 × 768 pixels; 1 pixel ~ 0.35 × 0.35 mm). The monitor was at a distance of ~65 cm in front of the participants centered at approximately eye level. Participant’s head was supported by a chin rest. Hand movements were performed on a graphics tablet (Intuos 4 A4, Wacom) placed on a table with the right hand holding a digitizing stylus. The distances covered by the stylus corresponded to the distances covered by the cursor displayed on the monitor. The vision of the hand during the movements was prevented by using a superstructure positioned above the tablet (see Fig. 1a). Perceptual judgments were made by pressing buttons of a computer mouse with the left hand.

#### Stimuli and trial procedure

All stimuli were displayed on a gray background. The main trial events are illustrated in Fig. 1a. Each trial started with a movement of the cursor controlled by the stylus movement (blue dot, ~ 2 mm in diameter) toward a starting position (dark-gray dot, 2 mm). During this movement the current score was shown in the upper middle part of the screen (in yellow) as well as a prompt to move the cursor to the start position (in light gray). After the starting position was reached three number-sign symbols were displayed for 1,000 ms in light gray in the middle of the screen. Then, an unframed square (~ 2.2 × 2.2 cm) consisting of small dark-gray dots was shown at the same position for 580 ms. The size of this stimulus corresponded to the mean size of the circles used as movement targets (see below). This size was chosen in order to induce a “neutral” focus size prior to target appearance (and not to prompt a small attentional focus probably induced by a smaller stimulus like a fixation cross). Following a blank screen lasting 60 ms, a dark-gray circle appeared in the middle of the display. This stimulus was visible for 350 ms and was the critical target that had to be hit by the movement cursor. A second circle appeared 67.7 mm to the left or to the right after 250 ms relative to the onset of the central circle for 100 ms. This lag of 250 ms was introduced to allow for unfolding of motor and attentional processes presumed to alter the apparent size of the central target before its site was measured. Then, the circles disappeared and the cursor appeared, together with a prompt to try to hit the target. During this movement, the cursor disappeared after it covered a half of the start-target distance. The participants had to press a stylus button when they reached the desired end point of the movement. After this button press, participants had to judge which circle was larger by the corresponding text shown in the upper middle part of the screen in green. The left/right mouse button had to be pressed when the left/right circle was larger (i.e., the relative positions of the circles were critical for the judgment, not whether a circle appeared in the middle or in the periphery). Finally, the movement target (and the prompt to hit the target) reappeared and the final cursor position was displayed for 500 ms. The movement target was now superimposed by four black unfilled circles, the radii of which corresponded to 0.25, 0.5, 0.75, and 1.0 of the target radius. These circles subdivided the target into four fields associated with different scores. Hitting the smallest field yielded 4 points, the next larger one yielded 3, the next 2, and the largest only 1 point. This final feedback display also contained the total score as well as points achieved in the current trial (indicated by an orange digit and a plus sign; see Fig. 1a).

An error display was presented, and the trial was repeated when the stylus left the starting position before the peripheral circle appeared or when the target movement was not finished within 2,500 ms after the cursor appeared on the screen.

Participants were asked to look at the center of the screen and not to move their eyes. Also, they were told to perform speedy movements in response to the peripheral circle (which appeared to be a more salient go signal than the small cursor) and to try to hit the target as often as possible. We also offered a bonus that scaled with the total score. Each paid participant received 8 Euro plus 1 Euro for at least 1,000 points, 3 Euro for 1,250 points, and 6 Euro for more than 1,500 points. One participant received an ungraded course credit of 105 minutes of participation (75 regular minutes plus a bonus of 30 minutes).

#### Design

The critical experimental variation was related to the location of the starting dot and thus to the magnitude of start-target distance to be covered to hit the target (“movement distance” hereafter). The starting position was either 39 mm (“small movement distance”) or 130 mm (“large movement distance”) below the central target stimulus (from center to center, see the upper right part of Fig. 1a).

To measure the expected changes in the perceived size of the central target we adopted a method of constant stimuli previously used in the research on attention and visual appearance (Carrasco et al., 2004; see also Carrasco & Barbot, 2019). One of the two circles served as a standard stimulus, the other was a test stimulus. The standard stimulus was always 22.4 mm in diameter. The size of the test stimulus varied from 87.5% to 112.5% of the standard size in nine steps. In one half of the trials the standard stimulus appeared at the central position, and the test stimulus was in the periphery. For the other half of the trials, the reverse was true. We refer to this factor as “type of central stimulus.” The order of all conditions was random in each block of trials.

There were four blocks of trials, including 144 trials each (16 repetitions for each movement distance, each type of central stimulus and each level of test stimulus). Before the main experiment started, participants performed 36 practice trials, which were not included in the analysis.

#### Data analysis

A proportion of trials in which the test stimulus was judged as larger was computed as a function of the test size, type of the central stimulus, and movement distance. A local model-free fitting procedure (Zychaluk & Foster, 2009) was used to fit these values with a psychometric function. The test size at which the test stimulus was chosen with a frequency of 50% (the point of subjective equality [PSE]) was determined for each type of central stimulus and each movement distance. The raw data have been made publicly available (https://osf.io/5x7h2/).

#### Hypothesis

An increase in movement distance was expected to increase the apparent size of the central circle that served as movement target. If this circle is the standard stimulus then a larger test stimulus should be required for the larger movement distance to perceive both stimuli as equal. In contrast, if the central stimulus is a test stimulus then a smaller test stimulus should be perceived as equal to the standard stimulus in the larger movement distance condition. In other words, the PSE was expected to increase for the large as compared with the small movement distance when the standard stimulus served as movement target, and to decrease when the test stimulus appeared in the center of the screen.

### Results and discussion

#### Hit rates

The target was less often hit when movement distance increased, *t*(15) = 5.91, *p* < .001. Mean hit rates were 0.99 (*SD* = 0.01) and 0.79 (*SD* = 0.14) for the small and large movement distance, respectively. This outcome indicates that the task was more difficult when movement distance was large than when it was small, and is in line with Fitts’ law (Fitts, 1954).

#### Size judgments

Mean PSE values and the corresponding judgment data are shown in Fig. 1b and c, respectively (see Fig. S1 in the Supplementary Materials for individual judgment data). The large movement distance was associated with a larger PSE than the small movement distance when the standard stimulus appeared in the middle of the display, and with a smaller PSE when the standard stimulus appeared in the periphery. This predicted interaction was significant, *F*(1, 15) = 4.76, *p* = .045, η_p_^2^ = .241. Pairwise comparisons revealed a significant difference between both movement distances for the central test stimulus, *t*(15) = 1.84, *p* = .043, and a marginally significant effect for the central standard stimulus *t*(15) = 1.35, *p* = .098 (one-tailed). These results confirmed the hypothesis that the target object is perceived as larger when movement distance increases, even though the effect was very small. Still, the results conceptually replicate our previous finding observed with a different experimental setup.

We also observed that circles presented in the periphery were judged as larger than circles presented in the center of the screen, *F*(1, 15) = 14.40, *p* = .002, η_p_^2^ = .490, *F*(1, 15) = .07, *p* = .790, η_p_^2^ = .005, for the main effect of movement distance. This side effect could appear surprising at first glance given that peripheral objects are usually perceived as smaller (e.g., Baldwin et al., 2016). However, as we recently demonstrated, this distortion is strongly modulated by the current locus of attention (Kirsch et al., 2020). In the present setup, the onset of the peripheral circle certainly caused a shift of attention toward the location of that circle. As a result, the usual effect direction was reversed (see also Experiment 2 in Kirsch et al., 2020). Importantly, this observation does not compromise the main finding, as such attentional shifts should be identical for both movement distance conditions.^2^

Please also note that the validity of the used method has been repeatedly approved (Anton-Erxleben et al., 2010; Anton-Erxleben et al., 2007; Carrasco et al., 2004; Kirsch et al., 2018; Liu et al., 2009; for a review of all controls, see Carrasco & Barbot, 2019) and that any systematic influences of a response bias or of eye movements that could potentially explain the results are very unlikely.

Overall, the results of Experiment 1 show that changes in action ability are accompanied by changes in size perception. This outcome is in line with our previous report (Kirsch et al., 2014) as well as with several related observations (see Introduction). Whether it arose from differences in attentional distribution between the action conditions has been explored in Experiment 2.

## Experiment 2

In Experiment 2, we basically retained the size judgment task, but replaced the motor task by another perceptual task aimed to induce changes in the size of attentional focus, similar to those which were presumed to cause apparent size changes observed in Experiment 1. In particular, we reasoned that in the more difficult large movement distance condition, more attention was allocated at the target center (associated with a maximum score) than when the movement distance was small, and thus hitting the target was rather easy. Accordingly, we introduced a letter discrimination task that forced the participants either to focus attention at the center of a to be judged circular stimulus (former movement target) or to spread it across a larger spatial area around this stimulus. Based on our previous results, the apparent size of this central stimulus was expected to increase for the small as compared with the large attentional focus (Kirsch et al., 2018).

### Methods

#### Participants

Thirty-two volunteers participated in Experiment 2. The sample included 23 females and nine males (*M* = 27 years, *SD* = 9). All participants reported to have normal or corrected-to-normal vision and to be right-handed. They received monetary compensation for their participation. None of them participated in Experiment 1.

The sample size was determined a priori and ensured a power of 0.95 for effect sizes of *d*_z_ = 0.6. Note that we used a similar procedure in one of our previous studies (Experiment 3 in Kirsch et al., 2018), in which we observed an effect size of η_p_^2^ = 0.263 for the critical interaction that would require a sample of 26 participants. In that study, however, the attentional distribution was constant across trials and blocks and varied between the participants. In the present study, in contrast, attentional conditions varied from trial to trial. This can be assumed to decrease their impact. In addition, in contrast to the present study, the locations of both target stimuli (circles) were constant and thus predictable in the previous study. Thus, more noise is expected in the present as compared with our previous study and a larger sample size appeared appropriate.

#### Apparatus

The apparatus was the same as in Experiment 1 except for the graphics tablet that was not used in Experiment 2. In Experiment 2, size judgments were made by pressing buttons of a computer mouse with the right hand. Responses to the letter discrimination task were accomplished by pressing arrow keys of the keyboard with the left hand.

#### Stimuli and trial procedure

**Fig. 2:**
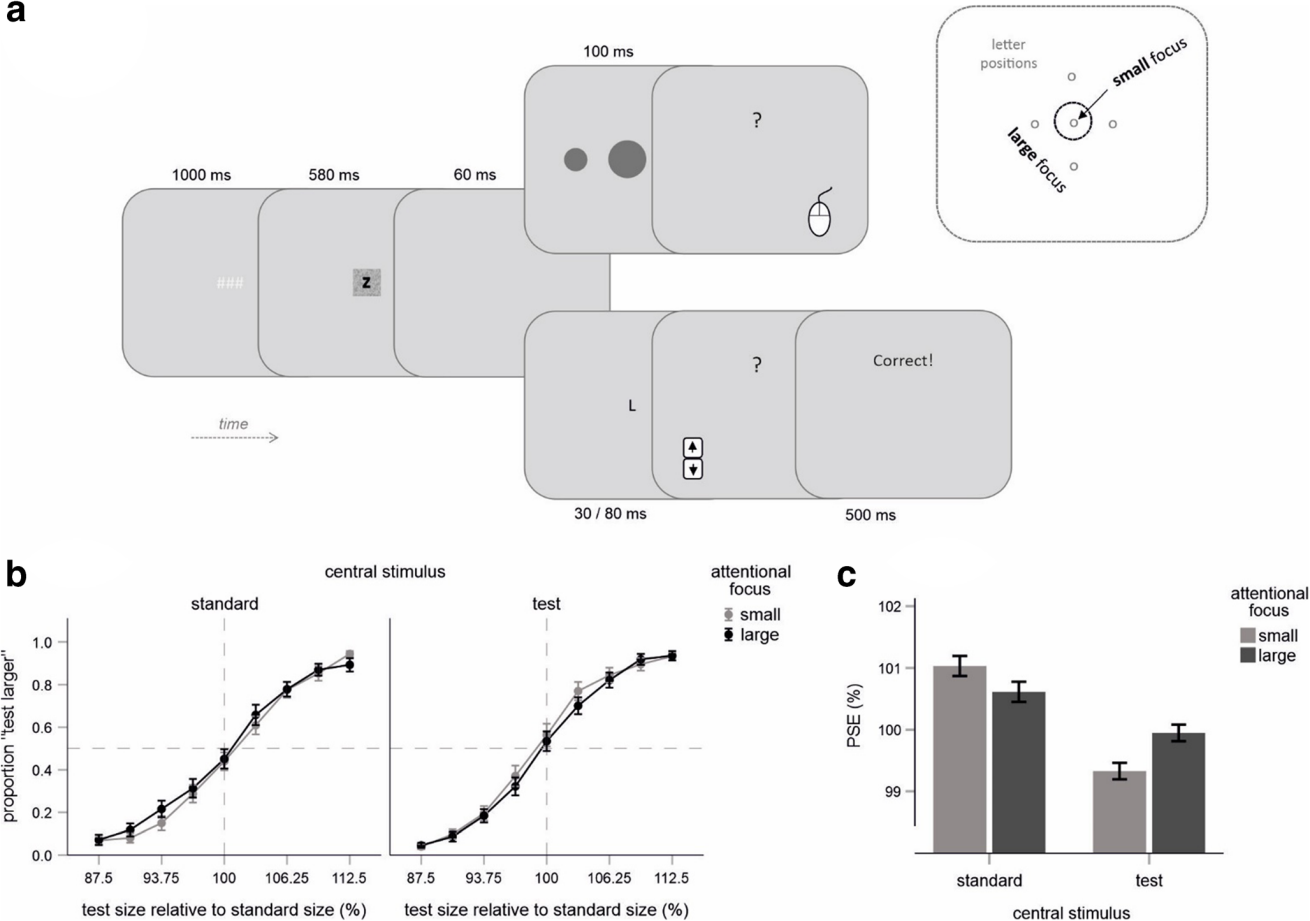
Experiment 2. **a** Main trial events and the critical variation of attention (upper right part). Stimuli are not drawn to scale. **b** Mean proportions of “test larger” judgments as a function of the type of central stimulus, attentional focus and of the size of the test stimulus. Error bars are standard errors indicting the variability across participants. **c** Mean PSE values as a function of the type of central stimulus and of attentional focus. Error bars indicate within-participants standard errors computed according to Cousineau (2005)

The main trial events are illustrated in Fig. 2a. The first three events were as in Experiment 1, except for the rectangle that was now superimposed either by a letter “Z” or by “U,” indicating the number and locations of target stimuli for the letter discrimination task (attentional cue; see also Design). In 50% of trials, one or four target letters (~2.5 mm in height) appeared for 30 or 80 ms, respectively, following the blank display. The letters “L” and “T” served here as target stimuli. The letter “Z” indicated that one target letter would appear, whereas “U” signalized four target letters. Following letter disappearance, participants were prompted (by a green question mark) to indicate the identity of either single letter when only one letter was presented, or the identity of the letter being in the majority in case of four letters. This judgment was done by pressing an arrow key of the keyboard (upper arrow key was assigned to “L,” lower arrow key to “T”). This key press was followed by a feedback display indicating whether the response was correct (“Correct!” in green), or not (“Wrong!” in red). In another 50% of trials, two circles were shown for 100 ms analogously to Experiment 1 (cf. Figs. 1a and 2a). Their size, location, and color were as in Experiment 1. In response to a question mark, participants had to indicate the larger circle by pressing a mouse button.

Participants were also asked to look at the center of the screen, not to move their eyes, and to be as precise as possible during the judgments. An error display was presented, and the trial was repeated when the participants mixed up the tasks (i.e., pressed a mouse button after letters or an arrow key after the circles).

#### Design

A critical experimental variation was related to the letter discrimination task (see Fig. 2a, upper right part). In one condition, the target letter was presented in the center of the display (“small attentional focus”). In another condition, four letters appeared above, below, left, and right to the center at a distance of 22.4 mm (“large attentional focus”). Participants were encouraged to use the attentional cue and to allocate attention accordingly (i.e., to focus attention at the center in response to “Z” and around the center in response to “U”). To measure the impact of this attentional manipulation on the perceived size of the central circle, the same method of constant stimuli was applied as in Experiment 1.

The order of all conditions was random in each block of trials. There were six blocks of trials including 144 trials each—size judgment task: 12 repetitions for each focus condition (i.e., for each attentional cue), each type of central stimulus and each level of test stimulus; letter discrimination task: 216 repetitions for each focus condition. Before the main experiment started, participants performed 36 practice trials, which were not included in the analysis.

#### Data analysis

Data analysis was performed in an analogous way, as in Experiment 1. Three participants had to be excluded from analyses. Two of them showed a very low discrimination performance in the size judgment task (see Participants 18 and 24 in Fig. S2 in the Supplementary Materials). The performance of another participant in the letter discrimination task was at chance level (51% on average; see Participant 16 in Fig. S2).

### Results and discussion

#### Letter discrimination task

The letter discrimination accuracy decreased for the large focus condition as compared with the small focus condition, *t*(28) = 4.81, *p* < .001. Mean values were 0.91 (*SD* = 0.11) and 0.79 (*SD* = 0.11) for the small and large focus, respectively. This outcome is in line with previous research and indicates that the large focus condition was more difficult (e.g., Müller et al., 2003).

#### Size judgments

Mean PSE values and the corresponding judgment data are shown in Fig. 2b and c, respectively (see Fig. S2 in the Supplementary Materials for individual judgment data). The small focus was associated with a larger PSEs than the large focus when the standard stimulus appeared in the middle of the display, and with a smaller PSE when the standard stimulus appeared in the periphery. This predicted interaction was significant, *F*(1, 28) = 5.00, *p* = .033, η_p_^2^ = .152. Pairwise comparisons revealed a significant difference between both focus condition for the central test stimulus, *t*(28) = 2.31, *p* = .014, and a trend toward significance for the central standard stimulus *t*(28) = 1.29, *p* = .104 (one-tailed).

In contrast to Experiment 1, circles presented in the periphery were judged as slightly smaller than circles presented in the center of the screen in Experiment 2 (see Fig. 2c). This often-observed tendency that we recently studied (Kirsch et al., 2020) was descriptively larger for the small movement distance, being, however, overall, not significant, *F*(1, 28) = .61, *p* = .442, η_p_^2^ = .021, for the main effect of type of central stimulus, and *ps* > .251 for (two-tailed) pairwise comparisons. One small but important difference between the current experiments and the related previous studies might be responsible for this outcome. In the present study, we did not use fixation crosses that can be assumed to increase the apparent size of the central target according to previous research (Kirsch et al., 2018; Kirsch et al., 2020). Using a larger fixation stimulus instead (i.e., square) could thus reduce the apparent difference between central and peripheral targets (at least in trials, in which the attentional cue was ignored). A descriptive trend toward an increase of this size eccentricity effect for the small focus condition, in which attention is supposed to be more in the center of the circle, seems further to support this view. Importantly, this observation (i.e., the lack of a significant size eccentricity effect) does not compromise the main finding of Experiment 2, as physical stimulation conditions were highly comparable for both attention conditions. Moreover, the main results of Experiment 2 can be considered as a conceptual replication of one of our previous experiments using only peripheral target stimuli (see Experiment 3 in Kirsch et al., 2018).

We also tested whether the effects observed in Experiment 1 and Experiment 2 differ. For this purpose, we assigned the level “large movement distance” of Experiment 1 to the level “small focus,” and the level “small movement distance” to the level “large focus,” and computed an analysis of variance (ANOVA) on the data of both experiments, including experiment as a between-subjects factor. This analysis revealed a significant interaction between type of central stimulus and focus size, *F*(1, 43) = 8.04, *p* = .007, η_p_^2^ = .158. The critical three-way interaction (Experiment × Type of Central Stimulus × Focus Size) was not significant, *F*(1, 43) = .009, *p* = .925, η_p_^2^ < .001. Thus, the manipulation of movement distance yielded basically the same effect as the manipulation of the attentional focus.

However, an Experiment × Type of Central Stimulus interaction was also significant, *F*(1, 43) = 5.92, *p* = .019, η_p_^2^ = .121, indicating differences in the judgments of central and peripheral stimuli between both experiments (all other effects were not significant, *p*s > .104). This effect indicated that the two experiments induced somewhat different changes in the focus of attention. The critical point here is that these changes are independent of the manipulations concerning the central stimulus (i.e., movement distance and focus size) as indicated by a nonsignificant three-way interaction. As mentioned earlier, a shift of attention toward the location of peripheral circles could well explain the larger estimates of those circles in Experiment 1 (see the “Results and Discussion” section of Experiment 1). As peripheral and central stimuli were simultaneously presented in Experiment 2, no such shift is expected, and using large stimuli for fixation diminished the usual size eccentricity effect (see below). These or similar factors that alter the relative size difference between central and peripheral stimuli are conceivably independent of the manipulations altering the apparent size of the central target stimulus under the present conditions.

Overall, the results of Experiment 2 conceptually replicate our previous observations and suggest that attended objects appear smaller with an increase in attentional spread (Kirsch et al., 2018). Moreover, they indicate that the modulation of size perception following changes in action planning observed in Experiment 1 is mediated by attentional mechanisms.

## General discussion

Previous research revealed manifold perceptual changes in the context of actions while their origin is still puzzling. The present study focused on the observation that changes in the ability to hit a target through action modulate the perceived size of the target object. By manipulating movement distance in a hitting task, and thus the hitting ability of the observer, we first demonstrated such a phenomenon in Experiment 1. In Experiment 2, we then omitted the action component (i.e., the hitting task), and instead manipulated the size of the attended spatial area under comparable stimulus conditions. The experimental variation of attention affected perception in the same way as the experimental variation of movement distance. These results support the idea that the observed changes in size perception accompanying changes in action ability originate from changes of spatial attention. In other words, attention rather than action seems to directly alter perception in the context of actions.

**Fig. 3:**
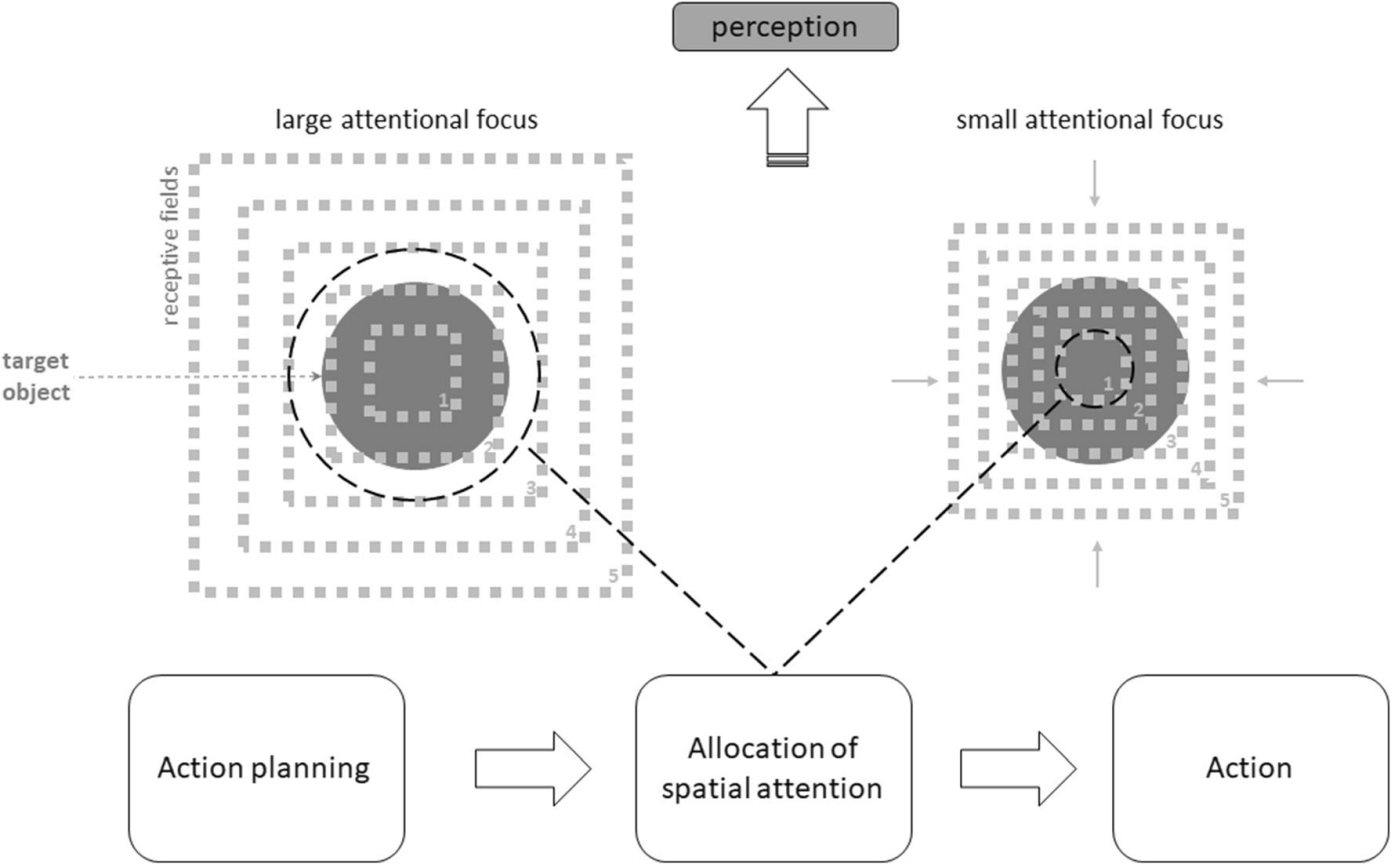
A crude sketch of the emergence of apparent size changes in the context of action under conditions of the present study. It is assumed that attention is more focused at the target object during action planning when the task is more difficult (i.e., when movement distance is large). This results in a stronger shift of receptive fields (RF, gray dots) toward the center of attention (indicated by thin arrows for the “small attentional focus”). As a result, the target object (dark-gray filled circle) stimulates additional RFs, which are outside the object when the focus is large (cf. dots numbered as “3”). Assuming that the same RFs code the same spatial locations, the target is perceptually magnified when the attentional focus is small (compared with the large focus)

Figure 3 provides an explanation for why focused attention entails a larger apparent object size than distributed attention. In essence, attending an object is assumed to cause a shift of receptive fields (RFs) of cortical neurons toward the focus of attention (cf. Anton-Erxleben & Carrasco, 2013; Baruch & Yeshurun, 2014; Carrasco & Barbot, 2019; Klein et al., 2016; Suzuki & Cavanagh, 1997; for neurophysiological evidence, see Anton-Erxleben et al., 2009; Klein et al., 2014; Womelsdorf et al., 2008). Importantly, the magnitude of this shift can be assumed to decrease with an increase in attentional spread (Baruch & Yeshurun, 2014; Kirsch et al., 2018; Klein et al., 2016). Thus, the same target object activates additional neurons, which code more distant locations when attention is focused than when it is distributed across a larger spatial area.

This mechanism could also be responsible for several related observations and resolve apparent discrepancies. For example, action success predicted larger estimates of target objects in several branches of sports, such as golf (Witt et al., 2008) or archery (Lee et al., 2012; see also Introduction). The present results indicate that this positive relation between apparent size and the current action ability arises basically because good performance is linked to a more focused mode of attention (see also Cañal-Bruland et al., 2011; Gray, 2013; Gray & Cañal-Bruland, 2015). This implies that more focused attention (at target object) is associated with better action performance (see, e.g., Castaneda & Gray, 2007). Whereas this assumption appears plausible under many natural conditions, this does not have to be always the case. For example, an observer could increase her effort and increasingly fixate the target object without success in spite of or just because her current action performance is disappointing. Such a behavior would explain why successful actions are sometimes associated with smaller judgments of target objects, like in our present and previous studies (Kirsch et al., 2014).

Previous research revealed four basic accounts for changes in perception following changes in action. First, action ability of the observer has been assumed to represent a type of reference scale for early sensory processing (Proffitt & Linkenauger 2013; Witt, 2011). Accordingly, changes in this reference are expected to result in changes of perception. Second, it has been suggested that perception and action share common cognitive representations (Hommel et al., 2001; Prinz, 1997). This approach predicts perceptual changes in the context of actions due to overlapping features of perceptual and motor codes (see, e.g., Section 4.3.2 in Hommel et al., 2001; Zwickel & Prinz, 2012). Third, action specific effects have been considered to result from sensory integration of visual and body-related signals by analogy to known interactions across other senses (Kirsch et al., 2017; Kirsch & Kunde, 2019). Fourth, it has been claimed that the reported effects are not perceptual in nature and reflect changes in participants’ judgment behavior (Durgin et al., 2012; Firestone & Scholl, 2016).

Here, we suggest that allocation of spatial attention is a crucial factor that affects object appearance in the context of action irrespective of the exact cognitive mechanism that alters attention. In essence, this approach does not contradict any of the previous theories, except for the judgment bias account (that denies any cognitive penetrability of perception). It merely stressed the critical level of processing at which perceptual changes could emerge. For example, sensory and motor processes could be merged during action planning according to a certain principle such as ability scaling, feature binding, or sensory integration. The critical point here is, however, that such a principle does not per se lead to changes in perception, it rather determines what and how is attended. Conceivably, the resulting changes in the characteristics of the current attentional focus (e.g., its location and distribution) are the real source of perceptual changes (see Fig. 3).

It has been presumed that an impact of action on perception facilitates adaptive behavior (e.g., Proffitt, 2006; Witt, 2011). At its heart, the present approach implies such an adaptive function in that changes in attention promote behavior that is optimal under given conditions. Action-specific perceptual changes, however, are merely a byproduct of adjusting attention and thus do not serve a specific function. This idea is consistent with several reports indicating that what and how people attend depends on what they currently plan to do (e.g., Baldauf & Deubel, 2010; Bekkering & Neggers, 2002; Deubel et al., 1998; Gutteling et al., 2011; Wykowska & Schubö, 2012; Wykowska et al., 2009). For example, the perception of object size is facilitated when a grasping movement is planned, whereas the perception of luminance is enhanced when a pointing movement is planned (Wykowska & Schubö, 2012; Wykowska et al., 2009).

These conclusions should be considered with caution due to at least two possible limitations. First, pairwise comparisons were partly nonsignificant in each experiment, indicating that perception was not affected when the test stimulus was in the periphery. We believe that this condition was slightly noisier than when the standard stimulus was in the periphery. Accordingly, the effect did not reach significance with the present sample size, while it did when the data of both experiments were combined (*p* = .04). Nevertheless, this issue needs additional work to be fully resolved. Moreover, one could argue that the “action effect” observed in Experiment 1 is unrelated to the “attentional effect” observed in Experiment 2 in spite of their similar magnitude and direction. For example, in Experiment 1, the target could be perceived as larger because larger movements signal a larger distance of the target (i.e., due to size constancy mechanisms; e.g., Epstein et al., 1961). We considered this assumption in our previous report (Kirsch et al., 2014, p.1761) and discuss it in more detail for another motor task (manuscript under review), but do not see inconsistency between size constancy and attention. In other words, size constancy could, in theory, be achieved through attentional mechanisms. There are also other related possibilities that can be explored in future studies. In addition, it would be interesting to more directly test whether an action task, such as a hitting task, in fact induces differences in the attentional distribution as we assume.

To sum up, Experiment 1 revealed changes in visual perception following changes in action. Experiment 2 indicated that this and related effects are mediated by changes in spatial attention accompanying action planning. These results extend previous research and provide new insights into how action affects perception.

## Supplementary Information

ESM 1 (DOCX 745 kb)

## Footnotes

1 Note that larger effort does not guarantee better hitting performance under the conditions of our previous study. In that study, participants were not informed about their final cursor position (they knew only whether they produced a hit or a miss). Thus, opportunities to improve hitting performance were rather limited. Accordingly, participants with lower scores could well be those who invested, without success, more effort to hit the target. In a similar vein, increased effort to hit a target from a more distant location could increase the hitting rate only to a certain extent due to biomechanical constraints and does not guarantee better hitting performance than in an easier condition with a smaller movement distance.

2 This effect is similar to the attentional repulsion effect (ARE)—apparent stimulus repulsion from the locus of attention as mentioned by a reviewer (e.g., Pratt & Arnott, 2008; Suzuki & Cavanagh, 1997). The contours of the peripheral target can here be considered as perceptually repulsed from the locus of attention (center of the peripheral target). Note that one supposed origin of the ARE (RF drift) is an integral part of logic of our experiments and explanations (see Fig. 3; see also Kirsch et al., 2018; Kirsch et al., 2020) and the size of the attentional focus can also modulate the ARE (Kirsch & Kunde, 2021). However, in the present experiment, this ARE like effect (i.e., increase in apparent size of the peripheral target) should be the same irrespective of the manipulations related to the central target (i.e., for both movement distances). Accordingly, the observed differences between both movement conditions cannot be attributed to peripheral attentional shifts.
