# Supplementary material for: Action affects perception through modulation of attention
Source: Atten Percept Psychophys. 2021 Mar 15;83(5):2320–30. doi: 10.3758/s13414-021-02277-2 (PMC8213557; doi:10.3758/s13414-021-02277-2)
Supplement: Supplementary file 1 — (DOCX 745 kb) [file 13414_2021_2277_MOESM1_ESM.docx]

**Supplementary materials**

**
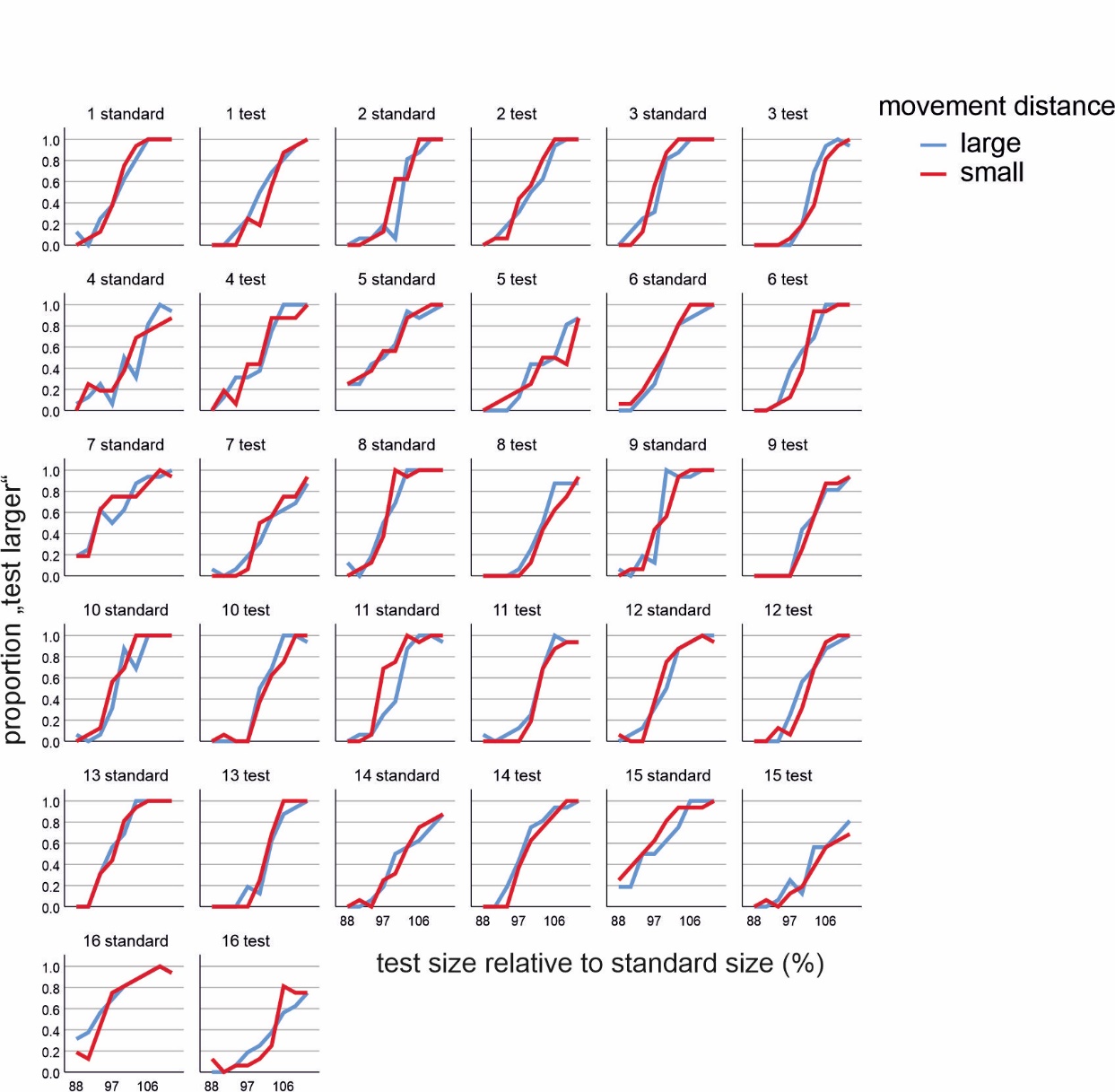
**

***Figure S1****. Individual data of the size judgment task for Exp.1. Values indicate the proportion of trials in which the test stimulus was judged as larger as a function of the type of central stimulus, movement distance and of the size of the test stimulus.*

**
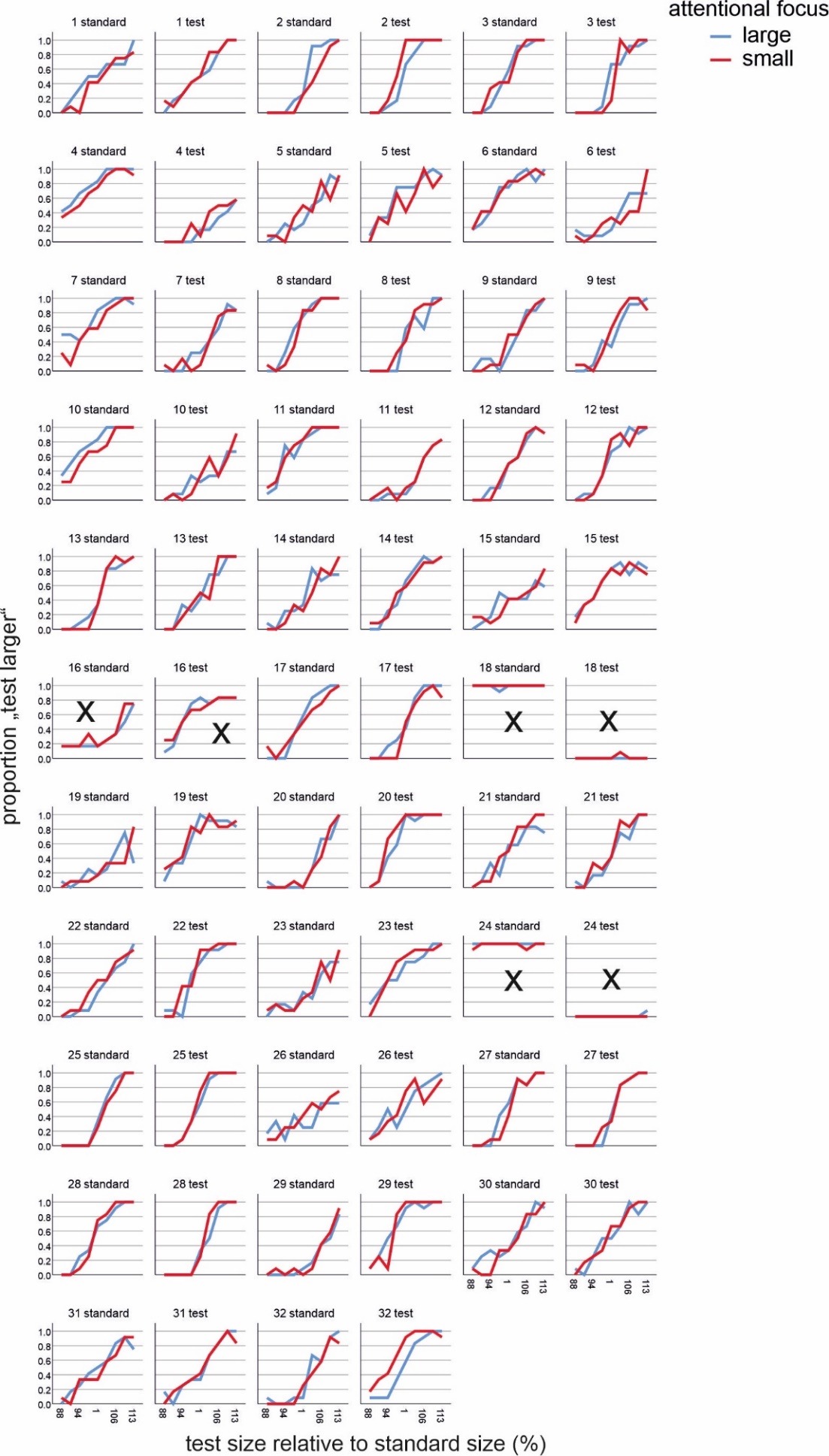
**

***Figure S2****. Individual data of the size judgment task for Exp.2. Values indicate the proportion of trials in which the test stimulus was judged as larger as a function of the type of central stimulus, attentional focus and of the size of the test stimulus. Xs indicate participants which were not included in the analyses.*
